# Lentivirus expressing shRNAs inhibit the replication of contagious ecthyma virus by targeting DNA polymerase gene

**DOI:** 10.1186/s12896-020-00611-4

**Published:** 2020-03-23

**Authors:** Leila Asadi Samani, Behnaz Saffar, Azam Mokhtari, Ehsan Arefian

**Affiliations:** 1 Department of Genetics, Faculty of Science, Shahrekord University, Rahbar Boulevard, Postal Box: 115, Shahrekord, Iran; 2 Biotechnology Research Institute, Shahrekord University, Shahrekord, Iran; 3 Department of Pathobiology, Faculty of veterinary medicine, Shahrekord University, Shahrekord, Iran; 4 Department of Microbiology, School of Biology, College of Science, University of Tehran, Tehran, Iran

**Keywords:** ORFV, Lentiviral plasmid, DNA polymerase, RNAi, TCID50, Real-time PCR

## Abstract

**Background:**

Contagious ecthyma or Orf is known as a zoonotic disease remains prevalently worldwide despite the application of some control strategies against it. RNAi particularly shRNA provides us with the chance to tackle this obstacle by an encouraging new approach. The current study indicates the design and experiment of third-generation lentivirus packaging systems delivering shRNAs to inhibit Orf virus (ORFV) replication and infection. Given the importance of DNA-pol gene in virus replication, in this study, three shRNAs against this gene were designed and cloned into lentiviral vectors to stabilize the expression of shRNAs. After producing lentivectors expressing ORFV-DNA– pol in HEK293T cells, the synthesized shRNAs were applied to downregulate viral replication and gene expression. The reduction in viral titer and RNA was evaluated by TCID50 test as well as real-time RT-PCR. The results were then analyzed in comparison with the control group.

**Results:**

Designed shRNAs significantly reduced virus yield approximately 90 to 97% and 96.8 to 99.4%, respectively compared to the control groups (cells infected with ORFV and infected with ORFV and scrambled vector) by TCID50 test. Real-time RT-PCR revealed a dramatic reduction in the expression of viral RNA approximately 99% compared to cells infected with ORFV and from 92.6 to 99%, respectively compared to cells infected with ORFV and scrambled vector.

**Conclusions:**

Therefore, it can be stated that RNAi is capable of being used as a potent therapeutically option against viruses like ORFV.

## Background

Orf Virus (ORFV), also known as sheep pox, ecthyma contagiosum, and contagious pustular dermatitis, is a *parapox virus,* which causes contagious ecthyma (CE) [1]. The virus not only affects the skin and mucosal membrane of ruminants but also as a zoonotic pathogen can infect humans, especially those working with animals [2, 3]. The skin lesions may lead to anorexia or starvation. Lesions on the udder and foot can prevent the offspring from feeding as well as transient lameness [3, 4].

While recent advances in research on Orf virus provide a new understanding of the basic mechanisms of the virus pathogenesis, none of the existing vaccines induces complete and long-term immunity; therefore, vaccinated animals can be re-infected. There is no effective vaccine against Orf disease in humans, sheep or goats [5].

With the ability to target each gene in the target tissue, RNAi treatments allow the development of faster and more cost-effective class of drugs. In addition, due to their ability to simultaneously target several genes, they may prove advantageous to cure complex and challenging diseases, especially cancer and viral infections [6].

siRNA or small interfering RNA has been proven to have some limitations: firstly, the relationship between the concentration of siRNA and its off-target effects; secondly, the decreasing concentration of siRNA in cell divisions. Thus, it is not possible to generate a stable cell line and knockdown target genes continuously and for a long time [7]. shRNA is an artificial RNA molecule with a hairpin loop that is transcribed by RNA polymerase in nucleus. The comparison of the efficiency of siRNA versus shRNA is difficult. However, shRNA is described to be somewhat more effective under similar production methods for both siRNA and shRNA. Compared to siRNA, shRNA is continuously synthesized in host cells, leading to longer gene silencing. The most important advantage with shRNA over siRNA is that it can be cloned in to a viral vector and enter into many cell lines [6]. Applying viral vectors potentially reduces the difficulties of transfecting cell lines; furthermore, because of their potential for stable gene expression, they are beneficial for in vivo studies [8]. Therefore, shRNA vector may be a practical tool for performing genes knockdown [9].

Lentiviruses are a subset of *retroviruses* that are capable of integrating into the genomes of both dividing and non-dividing cells in order to earn a long-term and stable expression of shRNAs [9]. Other benefits of lentiviruses include the following: broad tropism through pseudotyping, integration into the host genome to provide a durable expression of the encoded therapeutic protein, relatively easy production and negligible genotoxicity [10].

In the present study, having designed shRNAs against *ORF025* gene that encodes the DNA Pol, we generated lentiviruses expressing shRNAs in HEK 293 T cells in order to inhibit ORFV multiplication. Finally, we evaluated the rate of ORFV (Orf virus) replication in MDBK cells.

## Results

### Production of lentivirus expressing shRNA and challenging with Orf virus

**Fig. 1:**
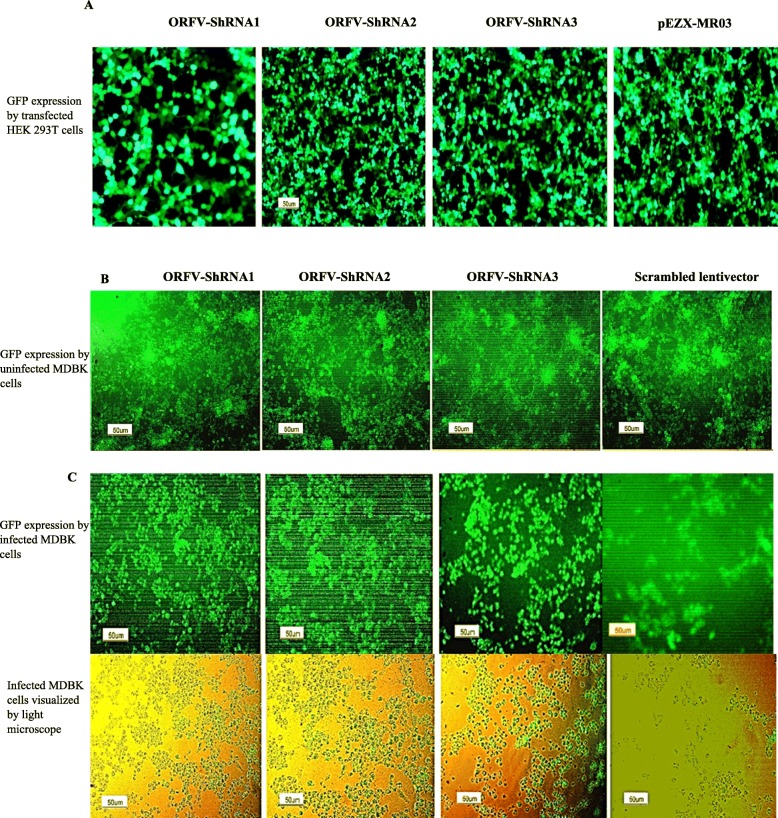
**a** GFP expression after Co-transfection of HEK293T cells: GFP expression by transfected cells with psPAX and pMD2.G as packaging vectors and pCDH carrying ORFV-ShRNA1, pCDH carrying ORFV-ShRNA2, pCDH carrying ORFV-ShRNA3 and pEZX-MR03 (as a mock), respectively. **b** Lentiviral ShRNA-GFP expression of uninfected MDBK cells: GFP expression by uninfected MDBK cells with lentivector expressing ORFV-ShRNA1, ORFV-ShRNA2, ORFV-ShRNA 3 and scrambled lentivector respectively. **c** GFP expression of ORFV infected MDBK cells with lentivectors: GFP expression by infected MDBK cells with lentivector expressing ORFV*-*ShRNA1, ORFV*-*ShRNA2, ORFV-ShRNA 3 and scrambled lentivector respectively and the same pictures with the light microscope

GFP expression by HEK293T and MDBK cells, which were co-infected with lentivectors expressing ORFV-ShRNA1, ORFV-ShRNA2, ORFV-ShRNA 3 and scrambled lentivectors, indicated that lentiviral vectors were successfully integrated and subsequently expressed in the cells (Fig. 1a and b). After the challenge, the survival rate of the cells infected with ORFV-shRNAs was higher than the infected cells with the scrambled lentivector, which indicates the lower development rate of the cytopathic effects of Orf virus in these cells (Fig. 1c).

**Fig. 2:**
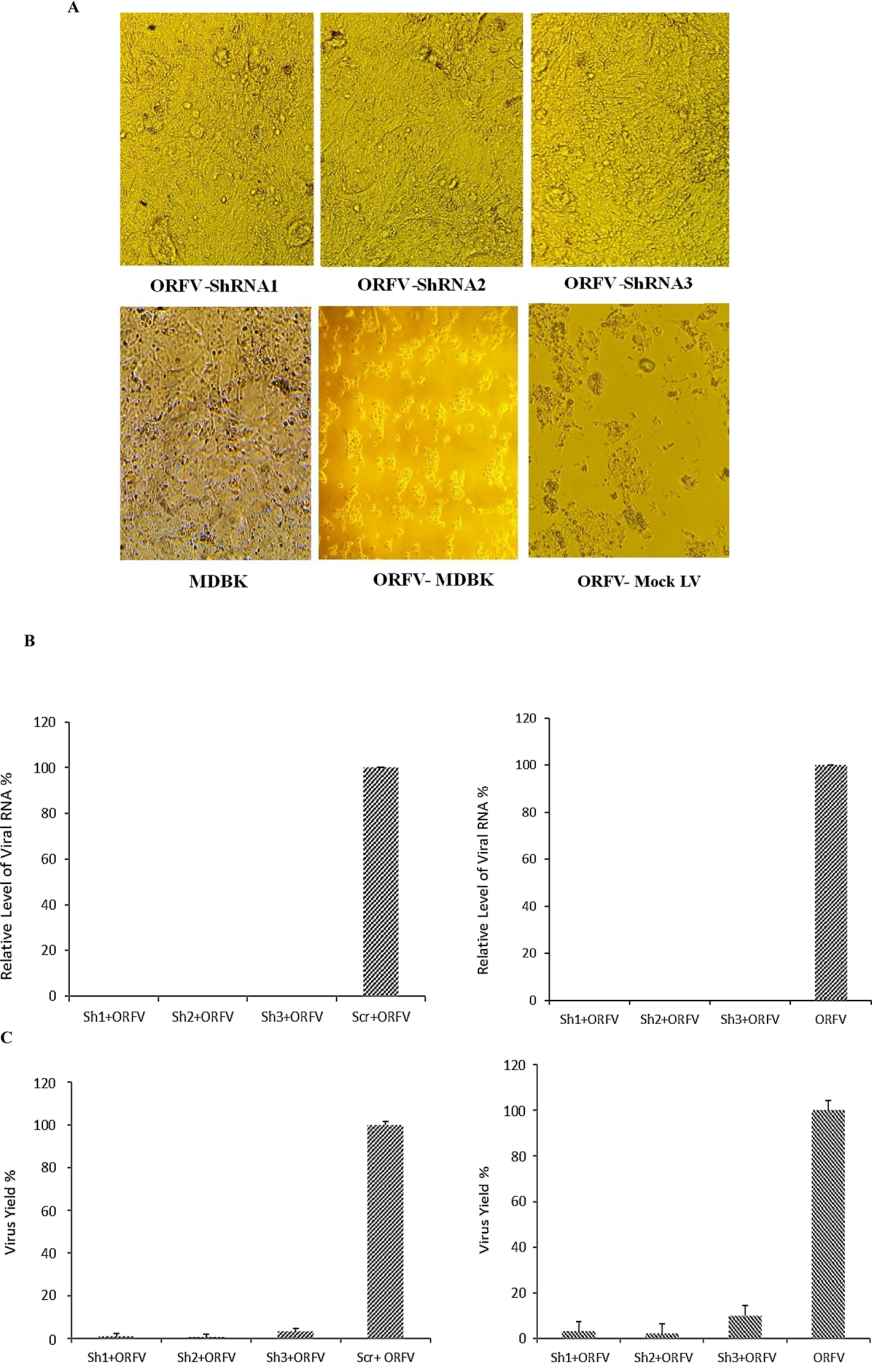
**a** Reduction of ORFV replication by shRNAs. The MDBK cells co- infected with lentivectors expressing shRNA and ORF virus. 72 h after infection, the CPE of ORFV was visualized with a light microscope. ShRNA 1,2 and 3 reduced cytopathic effects of ORFV and MDBK cells had a relatively normal morphology while in the wells infected by ORFV the cell morphology was changed. **b** Reducing ORFV by recombinant shRNA lentivirus: Extracted total RNA was used for real-time RT-PCR analysis. All the values were displayed in percentages of controls (cells infected with ORFV and those infected with Scrambled vector). The shRNAs 1, 2 and 3 noticeably decreased the expression of viral RNA compared to cells infected with ORFV and scrambled vector (Left chart) and compared to cells infected with ORFV (Right chart). **c** TCID50 assay: The titration of virus was detected at 48 h pi. The viral yields were measured by the ratio of TCID50 of expressed shRNAs to that of controls (cells infected with ORFV and those infected by Scrambled vector). Error bars show standard deviation of three independent experiments. The shRNAs 1, 2 and 3 reduced virus yield compared to cells infected with ORFV (Left chart) and compared to cells infected with ORFV and scrambled vector (Right chart)

*The inhibition* of ORFV *replication* using shRNAs was determined by EGFP expression in transduced cells with lentiviral vector and observed for CPE induced by ORFV. The results showed that cells infected with anti*-*ORFV shRNAs visibly reduced CPE 72 h after the infection, compared to those in the control group (MDBK cells infected with ORFV) (Fig. 2a). Seventy-two hours after the virus challenge, the real-time RT-PCR was used to estimate the effect of RNAi on ORFV replication and titration.

As depicted in Fig. 2b, shRNAs 1, 2 and 3 markedly reduced the expression of viral RNA in the studied cases compared to the control group (approximately 99% for three shRNAs compared to cells infected with ORFV and 99, 92.6 and 99% in the cells infected with ORFV and scrambled vector, respectively) (*P* < 0.05). Moreover, in the TCID50 test, the shRNAs 1, 2 and 3 significantly reduced virus yield in comparison to the control groups (approximately to 96, 97 and 90%, respectively in the cells infected with ORFV and to 99, 99.4 and 96.8% in the cells infected with ORFV and scrambled vector, respectively) (Fig. 2c). The results of CPE indicated a reduction in virus yields in each group.

### Reduction of ORFV DnaQ_like expression by lentivirus expressing shRNA

After the infection of MDBK cells, which were expressing ORFV DnaQ_like stably, and controls (MDBK cells infected with ORFV and those infected with mock vector) by lentiviral mediated shRNAs, the expressions of selected ORFV sequences were detected using real-time RT-PCR test.

**Fig. 3:**
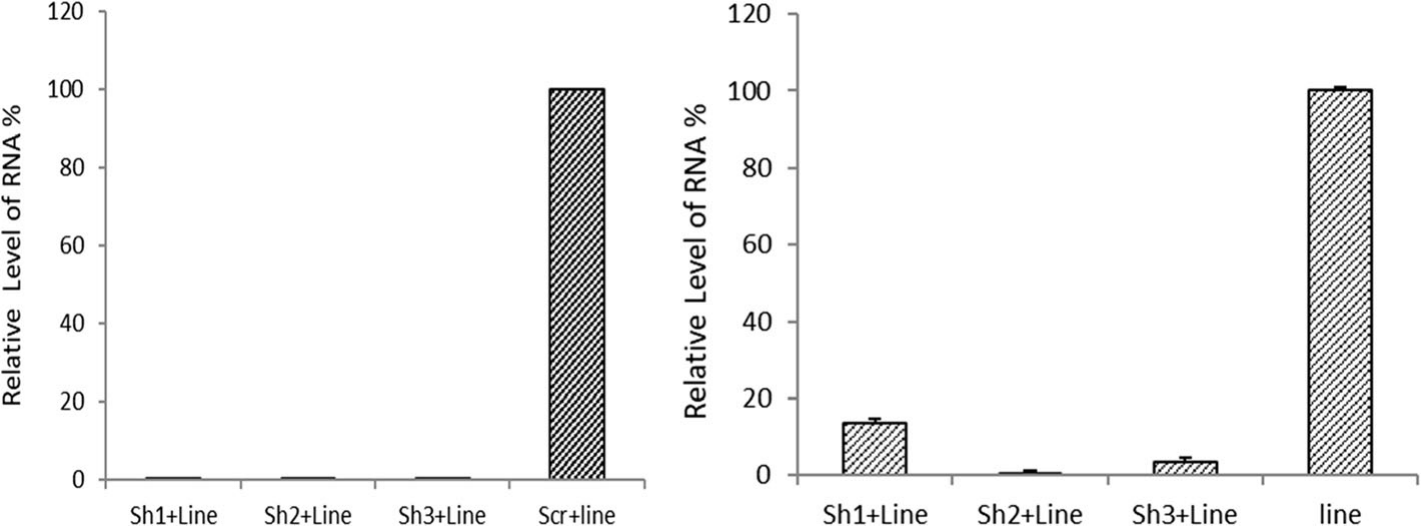
The inhibition of ORFV DnaQ_like expression by lentiviral expressing shRNA in MDBK cells. The total RNA was extracted and real-time RT-PCR was used for the determination of relative RNA. All the values were displayed in percentages of controls (cells infected with ORFV and those infected by Scrambled vector). The shRNAs 1, 2 and 3 markedly reduced expression selected gene compared to the control groups compared to cells without any infection (Left chart) and compared to cells infected by scrambled vector (Right chart)

As shown in Fig. 3, shRNAs 1, 2 and 3 significantly reduced the level of viral gene expression. The shRNAs 1, 2 and 3 markedly reduced the expression of the selected genes compared to the control groups (approximately 86.5, 99 and 96.5%, respectively compared to the uninfected cells and 99.9, 92.6 and 99.9% compared to that in the cells infected with scrambled vector, respectively). (*P* < 0.05).

## Discussion

Since ORFV is a zoonotic and highly contagious agent, with a global spread and expanding host range, its economic impact on the livestock industry is becoming more and more significant. The common control measures such as vaccination have not been successful in reducing its prevalence. Therefore, finding new and more effective treatment or control methods motivates scientists greatly. In Iran, unfortunately, the disease is prevalent in both traditional and industrial animal husbandry. Although some traditional, yet relatively effective, therapeutic measures are used, the treatment of acute ORFV infection is usually not effective in case of complications as well as secondary infections [11, 12].

The advantages of RNAi over classical vaccines and small-molecule drugs are its selective effectiveness against the target pathogen and its quick implementation on a large scale [13]. Recently, RNAi-based treatments against viral infections such as diseases caused by the members of Poxviridae have been reported [14]. Alkhalil et al. (2009) used siRNAs against 12 viral genes of Monkeypox virus and proved that RNAi has the potential to be used as a therapeutic measure for *Monkeypox* and other orthopox viruses [15]. Dave et al. (2006) utilized siRNAs against E3L gene of Vaccinia virus. By using two human cell types, namely HeLa and 293 T, their investigation indicated E3L-specific siRNAs as a powerful antiviral drug for small pox and related pox viruses [16]. In another study, Zhao et al. (2012) put four shRNAs against a region of *ORF095* gene, encoding virion core protein, and illustrated the widespread effect of DNA-based siRNA on inhibition of Goatpox virus replication [17]. In the case of ORFV, only one study was dedicated to RNAi and its application. Wang et al., (2014) applied siRNAs against DNA pol gene of ORFV via pMD-18 T vector. They reported approximately 73–89% less viral DNA in cells transfected with the siRNAs [18]. Despite many investigations to eradicate the disease, we used an RNAi-based approach to suppress ORFV replication in this study, due to the lack of specificity and side effects related to chemical drugs. In fact, thanks to the constant expression of the shRNAs delivered by lentivectors, the present study is the first survey to investigate the inhibitory effects of shRNA on ORFV replication. The third generation of lentiviruses is safer for therapeutic applications, since it contains only 10% of the viral genome sequence [19].

On the one hand, appropriate target transcripts should be selected when it comes to RNAi-based therapeutics. On the other hand, in the case of viruses, conserved sequences of genes in various virus strains need to be targeted. Hence, we selected the most highly conserved sequences, which have significant functions in replication.

Since shRNA is superior to siRNA in many respects, such as durability, fewer Off-target effects and lower dosage [20]. We constructed shRNA structures against ORF025-DNA pol gene that could effectively down-regulate virus replication. Eventually, lentiviral vectors will evolve as a beneficial tool to transfer genes constantly into desired cells with an abundant replicative potential. Indeed, the efficiency of lentiviral-mediated transduction of both non-dividing and dividing cells, as well as their potential for cell-specific pseudotyping - due to their flexible genome - allows for lentivectors to become a precious resource either in experimental platforms or therapeutic settings [21]. Based on a recent study, systemic in vivo application of lentiviruses, including viral quantification prior to intraperitoneal injection, and quantification of integrated provirus, is necessary for a sufficient lentivector mediated shRNA delivery [22]. In this study, we applied pCDH-CMV-MCS-EF1-cGFP-T2A-Puro vector as a third-generation lentivector to gain a stable expression of designed shRNAs, in order to achieve a long-lasting prevention of ORFV replication.

We did not find any studies on ORFV gene-expressing LVs, but a report on the preparation of an ORFV*-*based viral vector. In 2003, Fischer et al. cloned ORFVVEGF-E, D-and C-glycoproteins of pseudorabies in separate lentiviral plasmids, and subsequently co-transfected Vero cells with the generated vectors. Thus, a new viral vector based on Parapaxviruses was created [23].

Several cell lines expressing sub-replicons of some viruses have been developed to evaluate therapeutic and prophylactic therapies against viruses. For example, Basagoiti et al. (2009) cloned the target gene segments of Western Nile virus into appropriate plasmids and produced a cell line expressing the viral sub-replicons after transfection. This cell line was applied successfully to evaluate the chemical inhibitors of the WNV epidemic strains [24]. Kumar et al. (2006) found that lentivector pseudotypes that use RV-G instead of VSV-G are capable of delivering a specific RNAi induction in neuronal cells and significantly decrease the vector dose needed to reduce the target gene [25]. Wang et al. (2013) produced U6 shRNA induced by lentivectors expressing GFP, as a reporter protein. After cloning the nucleoprotein and matrix genes of Newcastle virus in the transfer plasmid and transfecting the cell lines suitable for expressing these genes, the shRNA molecules successfully inhibited the specific expression of these two genes and Newcastle Virus replication (90% inhibition of proliferation) [26].

Prior to the validation of shRNAs against ORFV, a cell line that expressed an ORFV sub-genomic replicon was produced for specific investigation of the target gene knockdown and to identify efficient shRNA molecules [27]. The data presented here showed that using this method is useful for screening functional shRNAs. Lantermann et al. (2007) found that the expression of early and late genes of vaccinia virus could be inhibited by siRNA. For specific evaluation of siRNA, they were evaluated in cell lines expressing vaccinia sub-genomic replicons [28]. Kilcher et al. (2014) applied RNAi to detect *AAA + ATPase D5* as an uncoating factor for vaccinia virus. In this study, the specific evaluation of the interfering molecules was performed through a sub-genomic replicon expressed by cells [29].

The focus of this work was in vitro study, but based on some research on transgenic animals that express shRNAs targeting disease agents, we concluded that antiviral therapy based on RNAi might be successful. The development of a goat that expressed shRNA targeting the prion protein, a viable calf expressing an anti-prion shRNA, the inhibition of porcine reproductive and respiratory syndrome infection by shRNAs and finally, production of transgenic mice expressing two anti-FMDV shRNA are four examples of moderate disease control or deletion of the target genes [13].

RNAi approaches in cell culture and rodent models as well as some animal studies have had promising results, but technical and market barriers must be taken into account before commercial applications of RNAi in livestock industry can be realized. Hopefully, our findings on shRNA-expressing lentivectors will challenge RNAi use in small and large animal models as well as its therapeutic potential for Orf disease. Elimination of viral toxicity, using appropriate controls and redundant shRNAs to ensure the accuracy of the results and to reduce any potential off-target effects are essential. Despite all obstacles, the convergence of a greater understanding of RNAi mechanisms, the detailed characterization of regulatory processes in animals and disease development, and the breakthrough in synthetic chemistry and genome engineering have provided exciting opportunities for RNAi use to enhance animal welfare.

## Conclusion

In conclusion, the *design* of *shRNA expressed by lentiviral vector illustrated the down –regulation by* real-time RT-PCR *and viral load methods.* Overall, ORFV*-*ShRNA2 had a better suppressive effect than two other anti ORFV*-* ShRNAs.

## Methods

### Design and preparation of shRNA molecules

In this study, first of all, the shRNAs targeting sequences ORF025-DNA pol gene were designed by means of three online design algorithm, namely: BLOCK-iTRNAi Designer (http://rnaidesigner.lifetechnologies.com/rnaiexpress/),WIsiRNA Selection Program (http://sirna.wi.mit.edu/home.php) and siRNA Wizard Software (www.invivogen.com/sirna-wizard). Having more precisely investigated the sequence and structural shRNA design rules, we selected the most potent ones [7, 30–32]. Apart from considering shRNA design tools, the proposed shRNAs were manually surveyed according to the parameters suggested by TomTuschl’s rules [30], Mcintyre et al. [31] and Taxman et al. [7].

A BLAST search needs to be performed in order to eliminate shRNAs having much homology with sheep genomic and transcripts database (http://blast.ncbi.nlm.nih.gov/), and fortunately no homologies were observed.

### Production of lentivector expressing shRNAs and ORFV- DNA polymerase

**Fig. 4:**
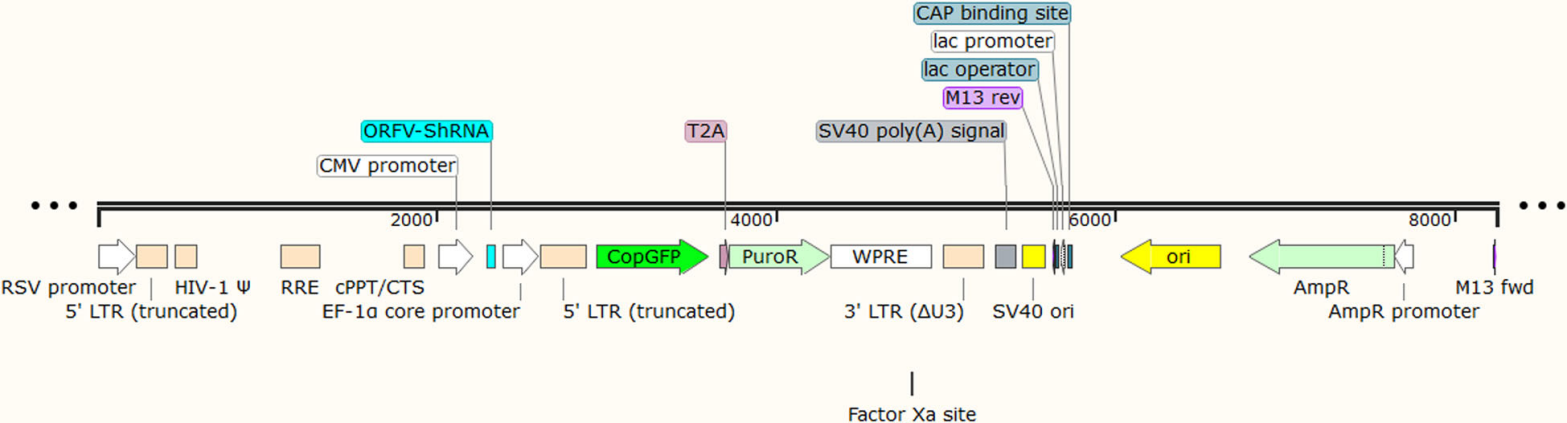
pCDH-CMV- shRNA-EF1-cGFP-T2A-Puro map

Three shRNA molecules were designed based on the ORFV-OV-SA00- DNA pol gene (accession number AY386264.1). Synthesized shRNAs were cloned separately into plasmids pCDH-CMV-MCS-EF1-cGFP-T2A-Puro to the downstream of the CMV promoter (which was kindly provided from Bonbiotech Company). The map of the vector is illustrated in Fig. 4. Furthermore, DnaQ_like exonuclease domain of ORFV-DNA pol gene was synthesized and cloned in the same lentivector. The lentivector used in this study, contained the CopGFP gene under the control of an EF1 promoter in order to pursue shRNA transfection efficiency. It is worth mentioning that the lentivector contained the gene of puromycin resistance cassette as well. All chemically-synthesized oligonucleotides were ligated into the lentivector which had been digested by *EcoR*I and *BamH*I. Ligated lentivectors were transformed into the *Escherichia coli* strain *DH5α.* After verifying the accuracy of cloning by sequencing, the accurate clones were used for transfection. As a mock (no shRNA GFP lentivector),we used pEZX-MR03 that contained the EGFP gene under the control of a CMV promoter lacking any significant shRNA sequences [33].

### Virus propagation

ORFV-SA00 standard strain was kindly provided by Dr. Asgari (Agricultural Research Service, United States Department of Agriculture, Greenport, New York). The titer of the virus stock was 8.91× 10^4^ TCID50/ml. ORFV-SA00 was used at 100TCID50 for in vitro infection experiments [18].

### Packaging of lentiviruses

In the present study, third-generation lentivirus packaging systems was applied in order to generate designed shRNAs and ORFV DnaQ_like expressing lentiviral vectors.

Having cultured HEK 293 T cells (ATCC Number: CRL-1573), we co-transfected the cells with a mixture of three vectors namely: 21 μg psPAX packaging vector,10.5 μg pMD2.G encoding the vesicular stomatitis virus glycoprotein (VSV-G) envelope and 21 μg pCDH (or pEZX-MR03 as a mock) transfer vector by means of Ca-Po4 reagent according to the bonbiotech (Iran) company’s guidelines. The co-transfection was performed in 10-cm plates similar to the conditions mentioned in manufacturer’s instructions in order to gain a confluency of approximately 70–80%.

Forty-eight and 72 h after transfection, the GFP expression in transfected cells was monitored and photographed by a fluorescent microscope. On the other hand, at the same time, the supernatant from these plates were collected and cleared by centrifugation (1000 g, 15 min) and were kept in − 70 °C until the infection of MDBK cells [34].

### Preparation of MDBK cells expressing ORFV DnaQ_like and its challenge with shRNA

To prepare MDBK cells expressing ORFV DnaQ_like, an amount of 10^5^ trypsinized MDBK cells were infected in a 6-well plate by lentiviral vectors (MOI = 0.8), diluted in 1 mL of DMEM medium supplemented by 10% FBS. The medium was changed with 2 ml of DMEM after 12 h of infection. At 48 h and 72 h after infection, MDBK cells were observed using a fluorescent microscope [35]. Infected cells were identified using RT-PCR. RNA was isolated from infected cells and then underwent a DNase treatment. Then, RT- PCR was carried out using the following primers: Forward: 5′- GGGACCGAGACAGTCAACTT − 3′ and Reverse: 5′- GGTCCCGTTGTTGTTGTTGA − 3′. The PCR thermal cycle programs include denaturation at 95 °C for 2 min followed by 30 cycles at 95 °C for 30 s, 52 °C for 30 s, and 72 °C for 30 s, followed by a final extension at 72 °C for 5 min. The positive (MDBK infected with ORFV) and negative (MDBK cells without any infection) controls were used in each test.

To infect MDBK cells expressing ORFV DnaQ_like, the cells (90% confluent) were infected in a 6-well plate by lentiviral vectors (MOI = 0.8), diluted in 1 ml of DMEM medium supplemented by 3% FBS. Twelve hours after infection, the medium was changed with DMEM supplemented by 10% FBS and 1X penicillin streptomycin (Sigma). The cells were re-infected after 12 h post changing medium. For each shRNA, three wells were considered [36].

### Infection /challenge of lentivirus expressing shRNAs with ORFV

3_*_10^5^MDBK cells (NBL-1; ATCC Number: CCL-22) per 6-well plates were seed and cultured in DMEM (Gibco, America, Catalog No. 116–12,800) with 10% FBS (Gibco, America, Catalog No. 106–10,270) 2 mM L-glutamine, 1X penicillin streptomycin (Sigma, America Catalog No. 116–12,800) as well as 2,5 mg/L amphotericin B at 37 °C with 5% CO2 incubator [37]. The next day, after removing the culture medium, lentiviruses were inoculated into wells. For each shRNA, three wells were considered. To achieve more infecting efficiency, 24 h after the first infection with lentivirus, the infection was repeated.

Uninfected and infected MDBK cells (48 h after infection) were monitored under a fluorescent microscope to evaluate GFP expression. In case of observing the appropriate GFP expression, the challenge with ORFV was performed. After 72 h, cells were monitored in terms of phenotypes and the development of CPE. MDBK cells infected with ORFV were considered as positive controls and MDBK cell infected with mock vector or without any viral infection were considered as negative controls [36].

### TCID50 assay

To determine the changes in CPE and reduction of ORFV titer, TCID50 was performed in 96-well plates with three replicates per dilution and in triplicate for each infection condition. Finally, viral titers were calculated based on the Spearman-Karber formula [38, 39].

### RT-qPCR

After 72 h of infection, total RNA was extracted from MDBK cells using the RNeasy mini kit (Qiagen, Crawley, UK). The on-column DNase digestion (Qiagen, Crawley, UK) was used to remove contamination of DNA. The *cDNA* was *synthesized* from *1* μg *total RNA* by reverse transcriptase enzyme.

Reverse-transcription performed by Superscript II (Invitrogen) and oligo (dT) for 1 h at 42 °C. RT-qPCR was performed using Power SYBR Green Mastermix (Applied Biosystems, Warrington, UK). The primers were designed using the GenScript real-time RT-PCR Primer Design web tool. The primer sequences were screened using a BLAST search to confirm its specificity and the PCR products were run on an agarose gel to confirm that products of the expected size were detected. The sequences of forward and reverse primers were as follows: Forward: 5´- GGGACCGAGACAGTCAACTT- 3´, Reverse: 5´- GGTCCCGTTGTTGTTGTTGA- 3´, *Bos Taurus* GAPDH Forward: 5′ - TGAGGACCAGGTTGTCTCCT- 3’and *Bos Taurus* GAPDH Reverse: 5′- CACCCTGTTGCTGTAGCCAAAT– 3′. The reactions were analyzed upon an iQ5 real-time RT-PCR detection system. The PCR reaction was started with 30 s minute *denaturation* at 95 °C. 40 cycles of 95 °C for 30 s, 52 °C for 45 s and 72 °C for 1 min were carried out. Cycle threshold (Ct) values were normalized to GAPDH, and a relative ORFV RNA level was done by the ∆∆Ct method [40]. The fold-change values relative to control were multiplied by 100 to obtain the reduction of percentage in ORFV replication. *Experiments were* performed in *triplicate.* The amplification was identified by *melting curve profile.*

## Data Availability

The data and materials used and/or analyzed during the current study are available from the corresponding author on reasonable request.

## Abbreviations

*BamH*I: Restriction endonuclease enzyme isolated from species *Bacillus amyloliquefaciens;*
BLAST: Basic Local Alignment Search Tool
Ca-Po4: Calcium orthophosphates
CMV: Cytomegalovirus
CopGFP: Green fluorescent protein cloned from copepod *Pontellina plumata*
CPE: Cytopathic effects
*DH5α*: E.coli cell engineered to maximize transformation efficiency
DMEM: Dulbecco’s Modified Eagle’s medium
DNA Pol: DNA polymerase
DNase: Deoxyribonuclease
E3L: Protein E3 gene of *Vaccinia virus*
*EcoR*I: A restriction endonuclease enzyme isolated from species *E. coli*
EF1: Elongation factor-1
EGFP: Enhanced GFP
FBS: Fetal bovine serum
GAPDH: Glyceraldehyde-3-phosphate dehydrogenase
GFP: Green fluorescent protein
HEK 293 T: Human embryonic kidney 293 T
HeLa: Henrietta Lacks’ ‘Immortal’ Cells
LVs: Lentiviral vectors
MDBK: Madin-Darby Bovine Kidney
MOI: Multiplicity of infection
ORFV: Contagious ecthyma virus
ORFV-SA00: Standard strain of ORFV
ORFV-shRNAs: shRNAs design against Orf virus-DNA pol gene that synthesized and cloned in the lentivector
RNAi: RNA interference
RT-PCR: Reverse transcription polymerase chain reaction
RT-qPCR: Quantitative reverse transcription PCR
shRNA: Short hairpin RNA
siRNA: Small interfering RNA
TCID50: 50% Tissue culture Infective Dose
U6: A type III RNA polymerase III promoter
VSV-G envelope: Vesicular stomatitis virus glycoprotein envelope
WNV: Western Neil’s virus
